# Clinical Holistic Medicine: Chronic Pain in the Locomotor System

**DOI:** 10.1100/tsw.2005.24

**Published:** 2005-02-24

**Authors:** Søren Ventegodt, Joav Merrick

**Affiliations:** ^1^ The Quality of Life Research Center, Teglgårdstræde 4-8, DK-1452 Copenhagen K, Denmark and The Scandinavian Foundation for Holistic Medicine, Sandvika, Norway; ^2^ National Institute of Child Health and Human Development, Office of the Medical Director, Division for Mental Retardation, Ministry of Social Affairs, Jerusalem and Zusman Child Development Center, Divisions of Pediatrics and Community Health, Ben Gurion University, Beer-Sheva, Israel

**Keywords:** quality of life, QOL, philosophy, human development, holistic medicine, public health, holistic health, holistic process theory, life mission theory, locomotor system pain, chronic pain, low back pain, Denmark

## Abstract

Most pains from the locomotor system arise due to involuntary, chronic tensions in the muscles or other tissues. When the patient is motivated, the pain is easily cured in most of the cases by using the tools of consciousness-based medicine, primarily therapeutic touch, conversation, and coaching the patient in a positive philosophy of life. The pains are often caused by “blockages” that may cause problems other than just pain. Often it turns out that the blocked areas develop actual physical damage over time: a slipped disk in the back, articular degeneration, or osteoarthritis when the cartilage is affected, can often be explained in this way. Apparently, the exact areas where the blockage is situated cause cellular problems, disrupting cellular order. The holistic process theory of healing and the related quality of life theories state that return to the natural state of being is possible, whenever the person gets the resources needed for existential healing. The resources needed are “holding” in the dimensions of awareness, respect, care, acknowledgment, and acceptance with support and processing in the dimensions of feeling, understanding, and letting go of negative attitudes and beliefs. The preconditions for holistic healing are trust and the intention for the healing to take place. Case stories of holistic treatment of patients with chronic back pain, low back pain, muscle problems, knee pain, and symptoms of rheumatoid arthritis are discussed with exercises relevant for patients with these conditions in the holistic clinic.

## INTRODUCTION

Pain is the most common reason for consultation with a physician[1]. Many of these pains are not cured by standard biomedical treatment[2], but become chronic with staggering negative health and economic consequences[3]. Pain is an extremely complex and difficult subject as it involves a whole range of aspects of the human being from the most bodily-physical to the most mental and spiritual[2,3,4,5]. Emotions seems to play an important role and pain is statistically connected to depression and anxiety[6]. The problem is that the pain will not go away, no matter what, and experiments with alternative methods like psychotherapy[7], relaxation techniques[8], and "spiritual healing"[9,10] seem only to have very limited effect.

When in severe or constant pain in the locomotor system, most patients go to see a physician and, in our experience, many patients can obtain partial or complete freedom from pain with consciousness-based holistic medicine. This is not the case when we are talking about terminal cancer or other terminal diseases, but even many of these patients can benefit from a holistic approach, as most patients have large hidden resources they can use. However, to obtain pain relief, the patient must be dedicated to develop into a more positive and happy individual who is less tense about him/herself and life. In our natural state, which holistic medicine helps us to recover, life is good and does not make the body hurt.

About 20% of the Danish population suffers from chronic pain and both children and adults are affected[11]. Often the pain of the locomotor system is in the neck, upper or lower back, shoulders, elbows, knees, hands, or feet. Closer analysis shows that pain is often located in particularly sensitive areas in muscles, tendons, connective tissues, joints, and bones. These areas are often called trigger points and are located in the parts of the body where we find most local tensions or blockages.

There are many theories about why we have those tensions. The most common explanation is that tension is due to tenseness, meaning a tendency to develop a "tense" personality. Instead of relaxing and being our own good self, we strain ourselves in many ways, consciously or unconsciously. We become tense while trying to fit, cope, and adapt in a different way than we actually are deep down in our nature. The tensions go all the way down to the deepest level of our existence. As we find this level (our soul or whole being) causal to the pain, this is the level we primarily address in our holistic medicine.

## CLINICAL HOLISTIC MEDICINE

The life mission theory[12,13,14,15,16,17] states that everybody has a purpose of life or huge talent. Happiness comes from living this purpose and succeeding in expressing the core talent in life. To do this, it is important to develop as a person into what is known as the natural condition, a condition where the person knows himself and uses all efforts to achieve what is most important for him. The holistic process theory of healing[18,19,20,21] and the related quality of life theories[22,23,24] state that the return to the natural state of being is possible whenever the person gets the resources needed for existential healing. The resources needed are "holding" in the dimensions of awareness, respect, care, acknowledgment, and acceptance with support and processing in the dimensions of feeling, understanding, and letting go of negative attitudes and beliefs. The preconditions for holistic healing are trust and the intention for the healing to take place. Existential healing is not a local healing of any tissue, but a healing of the wholeness of the person, making him much more resourceful, loving, and knowledgeable of himself, his needs, and his wishes. In letting go of negative attitudes and beliefs, the person returns to a more responsible existential position and an improved quality of life. The philosophical change of the person healing is often a change towards preferring difficult problems and challenges, instead of avoiding difficulties in life[25,26,27,28,29,30,31,32]. The person who becomes happier and more resourceful often also becomes more healthy, more talented, and more able to function[33,34,35].

## CASE STORIES FROM THE HOLISTIC CLINIC

Blockages encompass old feelings and can be dissolved once the feelings are liberated. It is difficult to explain the pain from "tensions", because we cannot just feel joint capsules or bones where blockages are sometimes located. Since it is often possible to provoke pain by pressing swollen or tense body areas without any muscle function (for instance, connective tissue above the sternum or fatty tissue above the low back), there must be some other explanation. Blockages are not tensions in the traditional sense, although they may be the cause of chronic muscle tensions.

> **Female, aged 30 years, with pain in fingers:** A 30-year-old woman presents with large swollen and tender finger joints. Signs of inflammation – sterile inflammation – and blood test results consistent with arthritis. The question is how the patient can strain her finger joints. By touch and massage ("manipulation") of the joints in her fingers, hands, arms, legs, feet and spinal column in holistic body therapy, an emotional tension was "released", as the patient becomes cross, angry and grumpy during the intense body therapy. Subsequently in gestalt therapy, the patient verbalised her feelings and finally understood who she is cross with and why. The arthritic symptoms – swelling and pain – vanished during the weeks that followed.

Clinical experience indicates that physical pain can be released when the patient can accommodate emotional pain. In holistic medicine, the theory concerning chronic pain in the locomotor system is supported by clinical experience. When patients are touched on tense and tender areas of the body, they often come into contact with old, unresolved problems and painful feelings from their personal lives.

> **Male, aged 39 years, with low back pain:** A 39-year-old man suffering from recurrent low back pain. Examination: his back is very tense and the facet joints in the low back have almost come apart in some places. Conversation revealed that he was suffering from anxiety. During gestalt therapy his anxiety was released, when he looked at his relationship with his authoritarian mother. Afterwards the back pain was resolved and did not return.

When the patients remembered and fully understood what went wrong, the blockage and the pain disappeared. This experience forms the basis of the Rosen method[36] and holistic body therapy.

## BLOCKAGES THAT ARE NOT RELEASED CAN CAUSE TISSUE DAMAGE

Blockages may cause problems other than just pain. Often it turns out that the blocked areas develop actual physical damage over time such as a slipped disk in the back, articular degeneration, or osteoarthritis when the cartilage is affected. Apparently, the exact areas where the blockage is situated cause cellular problems, disrupting cellular order. This disruption is reversible. The renowned international medical textbook, *Harrison's Internal Medicine*, states that even degenerated cartilage, as it occurs in osteoarthritis, is sometimes seen on X-rays to have regenerated. That is the sort of healing effect that may occur once blockages around the joint are released. We have performed pilot studies in groups of pain patients with a regime combining life philosophy, psychotherapy, and body therapy. Some of the participants had severe physical disabilities and damage. The preliminary results of treating patients with different diagnoses indicate that, in general, working on the release of blockages in the body can be an effective way of abolishing chronic pain.

> **Female, aged 38 years, with chronic back pain:** Conversation: She has had chronic back pain for over 12 years. On examination: Very tense in back muscles between iliac

> crest and column. She believes that the pain has a psychological explanation. She wants to work on herself to get rid of pain and arrive highly motivated. Subjective: She explains that her younger brother is mentally ill – anxiety, depression and attempted suicide – and starts crying. There have also been problems with the parents. “I am the one who has to cope with it all,” she says when we talk about self-esteem, “but I’m not there.” She can see that she is suppressing her feelings to make herself strong, and that this is how she avoids being present in life. She can feel that she does so by tensing the low back, exactly where it hurts. EXERCISE for next time: “Find out where you are – where did you go?” PLAN: When she is ready, she should have physiotherapy with massaging of back.

Once one has seen how people suppress their lives to adjust to people around them, this pattern becomes easily recognizable in the patient. This woman has completely opted out of her own existence to be as sensible as she felt she needed to be in order to survive at the social level. The problem is that in the middle of all her control and sense, she is not at all present as a human being with feelings and presence. She is hiding; she has fled life. Unfortunately, the feelings of anxiety and discomfort are parked in her upper and lower back as tension, and that gives her great problems. Once she learns to apply herself better, her chronic back pain will go away. This typically takes six sessions over 3 months of exercise and assignments.

> **Male, aged 51 years, with chronic back pain:** His back snapped following construction work. Pain and some throbbing down into the legs. Saw a chiropractor twice with no effect. On examination: no significant back pathology (in spite of X-rays), swollen and tender at sacroiliac joints and piriformis, which are massaged with some effect. The patient believes that this is old damage that cannot be helped. Very tense between shoulder blades, where the back has not yet “snapped”. Manipulation not possible, because of muscle tension. He will come back for treatment of this region and further assessment in 14 days. EXERCISE: Pelvic exercises and examine at home why he is so tense.

The idea that the body is a machine that may break down irretrievably is widespread. If you understand the body the way we do, as a dynamic colony of cells controlled by the information flow, you would have to smile wryly at remarks of the type made by this patient: “Old damage that cannot be helped.” When people “break their back”, they believe that the back is almost torn apart, never to be put together again, like a trunk that has broken. In general, it is quite astonishing how little patients know about the anatomy of the back. Fortunately back problems can usually be resolved when the patient finds out why he or she is tense and tackles the underlying emotional issue. Once the patient has assumed responsibility for this, it is usually possible to manipulate the vertebrae back into place. Actually, vertebrae and joints, which can be quite distorted and shifted in relation to each other, will usually fall spontaneously into place once the muscles relax. Often a 10-minute talk on the couch before the manipulation will do the trick. It is peculiar how you tear and press and nothing happens, then you talk for a while and snap!…. it yields to the smallest pressure and the pain goes away.

> **Male, aged 61 years, with chronic back pain for 20 years:** Immigrant presenting with back pain dating back 20 years. He has taken analgesics over the years. Now distinct tenderness (doorbell) corresponding to about L2. No other symptoms. Says chiropractor could not help. Remarkably rigid gait. Needs to exercise his back. Instructed in back exercises. Advised to swim once or twice a day. The effect will take a while to appear, perhaps months or years. But that is the only way out of the problem.

Cultural differences and language barriers mean that we cannot apply a psychosomatic approach, but must try a combination of exercise and remarks to strengthen the patient's physical confidence. This is harder work, painful and sad.

> **Female, aged 24 years, with low back pain:** Presents with low back pain corresponding to right sacroiliac joint [between os sacrum and pelvis]. On examination: Articular tenderness consistent with /sprained left sacroiliac joint/. Very tense in scalenus muscles, neck, shoulders and particularly the center of the back, where large groups of muscle are organised as tethered and tense muscular masses causing /enlarged kyphotic curve/ that is compensated by /enlarged low-back lordosis/, which is presumably the cause of the low back pain. So the problem lies in the tensions. We talk about where they come from and the patient mentions her parents' divorce. EXERCISE: Tilting and rotating the pelvis ("pelvic rotations" [good exercise in which the patient swings the abdomen around in a horizontal circle in a diameter of about one metre]). Can return if the problem persists.

Young people almost never process their parent's divorce. It is quite surprising how little it takes to start relevant processing. When the patient has some exercises that gently release the blockages from the body, combined with one that supports processing of the emotional trauma, a clear and motivated individual can rid himself of a chronic, low back condition fairly rapidly. Other times it may bother him for years.

> **Male, aged 61 years, with severe pain in thigh following intercourse:** Left thigh very sore, daily attacks corresponding to posterior side of thigh, a piercing, throbbing, hammering pain, sometimes the whole day, sometimes up to a whole week after intercourse. Pain brought on by flexing of hips. Rectal exploration: no tumours, no tenderness, nothing abnormal. Free hernial orifices. Muscle tension corresponding to quadriceps femoris and psoas major muscles. The familiar pain is induced by pressing on these muscles. /Muscle pain/ – does not want analgesics. He is instructed to try to breathe instead of tensing up and shutting off at attacks. Can come back if pain persists; then we shall have another talk about medication.

When the familiar pain is induced by pressing on certain muscles, the patient knows that we are aware of his pain and why he is in pain. This means that the patient should be given a confrontational exercise where he can use his breathing (breathe in deeply and into the abdomen and then exhale) to be present when he is cramped and sore. That can often deal with the problem. The focused consciousness, where you become more familiar with yourself and your body, is a brilliant tool for the patient to use.

> **Male, aged 30 years, with knee pain:** Presents with problems in left knee: hurts after he has been sitting for 10 min. On examination: oedema in legs and around the knee. Talk about job, using the body, etc. We talk about him getting away from cyberspace (spending hours on the Internet) and finding a girlfriend – getting out into the world where his body is. His body would benefit from that. Guidance.

Get a life! The body does not much like sitting in front of a computer screen day in, day out, completely denied by its owner.

> **Female, aged 55 years, with muscle problems:** QOL conversation: Problems with muscles, tendons and joints for 12 years. Pain and reduced strength, particularly in the arms, the left being worse. Reduced mobility of shoulder. On examination: very small muscles that are chronically tense and resemble "steel strings" in shoulder girdle. Seems to be as frozen as an ice-lolly in the upper part of the chest. We talk about feelings, which in my opinion do not come alive in the patient. It is as if she is very warm, but has decided to be cold. PLAN: The patient is adept at avoiding emotional pain, so we prescribe Rosen method 6 times every 14 days, which supports the patient in registering all those old, painful feelings that she has denied. EXERCISE: Welcome all negative feelings. Be with them, inside them, allow them space. Come back in three months.

Some patients are really hard to get through to. It is good for them to lie on a couch and feel, feel, feel, until one day they finally realize what it is all about. Then they can come back to our clinic and unable us to move on.

## DISCUSSION

The case stories show, in many different ways, how the physician assuming the holistic medical perspective addresses the physical pain. Not all patients welcome this perspective, as it inevitably ends with the patient going through often severe emotional difficulties, confronting negative attitudes and shadowy aspects of their own existence. A lot of trust in the physician is necessary before the holistic toolbox[37] can be used freely. To win the trust, the physician needs to care for his patient and express this care, which is difficult for many physicians with traditional biomedical training. When the trust is won, the patient must accept the holding and support and in this privileged state, the patient can heal. Therapeutic touch[38] is often helpful when trust is obtained.

Often the problems are more of a social type than of a somatic type[39]. What might look easily treatable turns out to be very difficult, unless the social circumstance is handled successfully through coaching of the patient to help him create value and mobilize resources and talent in the social relationships. Sometimes, the physician must face severe problems connected with interpersonal chemistry; this is the case when he has something to learn from his patient because he is not sufficiently developed in the existential aspects relevant for the holistic treatment of this special patient[18].

Most pain from the locomotor system arises because of involuntary, chronic tensions in the muscles or other tissues. When the patient is motivated, the pain is easily cured in most of the cases by using the tools of consciousness-based medicine, primarily therapeutic touch, conversation, and coaching the patient in a philosophy of life that supports life and a constructive behavior. As previously discussed, the holistic process theory of healing[18,19,20,21] and the related quality of life theories[22,23,24] state that the return to the natural state of being is possible whenever the person gets the resources needed for existential healing. The resources needed are holding in the dimensions of awareness, respect, care, acknowledgment, and acceptance with support and processing in the dimensions of feeling, understanding, and letting go of negative attitudes and beliefs. The precondition for the holistic healing is deep trust and the intention of the healing to take place. The holistic cure for chronic pains and discomfort can be used on almost all patients independent of the site and affected tissue, but the efficiency of the cure has yet to be documented clinically in larger series of patients.

## ACKNOWLEDGMENTS

This study was supported by grants from IMK Almene Fond. The quality of life research was approved by the Copenhagen Scientific Ethical Committee under number (KF)V.100.2123/91.

## BIOSKETCHES

**Søren Ventegodt, MD,** is the director of the Quality of Life Research Center in Copenhagen, Denmark. He is also responsible for a Research Clinic for Holistic Medicine in Copenhagen and is a popular speaker throughout Scandinavia. He has published numerous scientific or popular articles and a number of books on holistic medicine, quality of life, and quality of working life. His most important scientific contributions are the comprehensive SEQOL questionnaire, the very short QoL5 questionnaire, the integrated QOL theory, the holistic process theory, the life mission theory, and the ongoing Danish Quality of Life Research Survey, 1991–94 in cooperation with the University Hospital of Copenhagen and the late professor of pediatrics, Bengt Zachau-Christiansen, MD, PhD. E-mail: ventegodt@livskvalitet.org. Website: http://www.livskvalitet.org

**Joav Merrick, MD, DMSc,** is professor of child health and human development affiliated with the Zusman Child Development Center, Division of Pediatrics and Community Health at the Ben Gurion University, Beer-Sheva, Israel; the medical director of the Division for Mental Retardation, Ministry of Social Affairs, Jerusalem; and founder and director of the National Institute of Child Health and Human Development. He has numerous publications in the field of child health and human development, rehabilitation, intellectual disability, disability, health, welfare, abuse, advocacy, quality of life and prevention. Dr. Merrick received the Peter Sabroe Child Award for outstanding work on behalf of Danish Children in 1985 and the International LEGO-Prize ("The Children's Nobel Prize") for an extraordinary contribution towards improvement in child welfare and well being in 1987. E-mail: jmerrick@internet-zahav.net. Website: www.nichd-israel.com
